# Putative Autoantigen Leiomodin-1 Is Expressed in the Human Brain and in the Membrane Fraction of Newly Formed Neurons

**DOI:** 10.3390/pathogens9121036

**Published:** 2020-12-10

**Authors:** David W. Nauen, Michael C. Haffner, Juyun Kim, Qizhi Zheng, Hao Yin, Angelo M. DeMarzo, Vasiliki Mahairaki, Carlo Colantuoni, J. Geoffrey Pickering, Tory P. Johnson

**Affiliations:** 1 Department of Pathology, Johns Hopkins School of Medicine, Baltimore, MD 21287, USA; dwnauen@jhmi.edu (D.W.N.); mhaffne4@jhmi.edu (M.C.H.); qzheng3@jhmi.edu (Q.Z.); ademarz@jhmi.edu (A.M.D.); 2 Department of Neurology, Johns Hopkins School of Medicine, Baltimore, MD 21287, USA; juyun.kim2856@gmail.com (J.K.); vmachai1@jhmi.edu (V.M.); ccolantu@jhmi.edu (C.C.); 3 Robarts Research Institute, Western University, London, ON N6A 3K7, Canada; hyin@robarts.ca (H.Y.); gpickering@robarts.ca (J.G.P.); 4 Department of Neuroscience, Johns Hopkins School of Medicine, Baltimore, MD 21287, USA; 5 Institute of Genome Sciences, University of Maryland School of Medicine, Baltimore, MD 21287, USA; 6 Department of Medicine, Western University, London, ON N6A 3K7, Canada; 7 Department of Medical Biophysics, Western University, London, ON N6A 3K7, Canada; 8 Department of Biochemistry, Western University, London, ON N6A 3K7, Canada

**Keywords:** nodding syndrome, leiomodin-1, autoantibody, CNS, neuron, membrane

## Abstract

Nodding syndrome is a pediatric epilepsy disorder associated with *Onchocerca volvulus* infection, but the mechanism driving this relationship is unclear. One hypothesis proposes that parasite-induced immune responses cross-react with human leiomodin-1 resulting in immune-mediated central nervous system (CNS) damage. However, as leiomodin-1 expression and epitope availability in human neurons remains uncharacterized, the relevance of leiomodin-1 autoimmunity is unknown. Leiomodin-1 transcript expression was assessed in silico using publicly available ribonucleic acid (RNA) sequencing databases and in tissue by in situ hybridization and quantitative polymerase chain reaction. Abundance and subcellular localization were examined by cell fractionation and immunoblotting. Leiomodin-1 transcripts were expressed in cells of the CNS, including neurons and astrocytes. Protein was detectable from all brain regions examined as well as from representative cell lines and in vitro differentiated neurons and astrocytes. Leiomodin-1 was expressed on the membrane of newly formed neurons, but not neural progenitor cells or mature neurons. Importantly, leiomodin-1 antibodies were only toxic to cells expressing leiomodin-1 on the membrane. Our findings provide evidence that leiomodin-1 is expressed in human neurons and glia. Furthermore, we show membrane expression mediates leiomodin-1 antibody toxicity, suggesting these antibodies may play a role in pathogenesis.

## 1. Introduction

Nodding syndrome is a form of pediatric epilepsy that has occurred in an epidemic fashion in regions of Northern Uganda, South Sudan, and Tanzania [1,2,3]. Affected patients initially present with atonic seizures, and other forms of seizure may be associated with disease progression [1,3,4,5,6]. Extensive epidemiological investigations have revealed a consistent association with the parasite *Onchocerca volvulus*, the etiologic agent of onchocerciasis [1,2,5,7,8] and cohort studies have revealed a temporal and dose-dependent correlation between *O. volvulus* infection and subsequent development of epilepsy in pediatric patients [9,10]. Notably, while the parasite infects individuals of any age, nodding syndrome is observed only in pediatric patients, with an age of onset between five and 15 years [2,3]. Adults with heavy parasite loads do not appear to be at an increased risk for the development of this syndrome or other forms of epilepsy [3,11]. As *O. volvulus* is not thought to enter the central nervous system (CNS) [1,5,7], the underlying mechanism driving the relationship between *O. volvulus* and nodding syndrome has remained unclear. 

One hypothesis is that *O. volvulus-*associated epilepsy, including nodding syndrome, is due to CNS damage caused by immune responses to the parasite. Several autoimmune epilepsies have been described in which the immune system targets neuronal surface proteins and disrupts neuronal function. Examples include those involving N-methyl-D-aspartate receptor (NMDAR), α-amino-3-hydroxy-5-methyl-4-isoxazolepropionic acid (AMPA)receptor (AMPAR), and voltage-gated potassium channel (VGKC) [12]. An immune response targeting the parasite was recently described to cross-react with human leiomodin-1 [13]. 

Leiomodin-1 is a member of the tropomodulin family of proteins and functions as an actin nucleating protein in smooth muscle cells [14]. While previously reported as having limited tissue expression, leiomodin-1 was found to be expressed in neurons differentiated in vitro [13]. Furthermore, antibodies to leiomodin-1 were toxic to iCell human neurons, a mixture of post-mitotic neural subtypes in culture [13], suggesting that these antibodies may play a role in the disease process. However, as leiomodin-1 is thought to be restricted to intracellular expression, and antibodies to intracellular antigens typically do not cause toxicity, the mechanism by which leiomodin-1 antibodies mediate neurotoxicity or might contribute to the development of nodding syndrome is unclear.

Here, we sought to determine if leiomodin-1 is a relevant human CNS autoantigen. We examined the expression of leiomodin-1 in the human CNS and evaluated the cellular and subcellular expression of leiomodin-1 in human differentiating and mature neurons and glia in vitro. We found that leiomodin-1 is expressed in neurons and glia from the human CNS and that leiomodin-1 was present in the membrane fraction of newly formed neurons. We therefore assessed neurotoxicity induced by antibodies to leiomodin-1 in in vitro differentiated neurons at different maturation states. We found that antibodies to leiomodin-1 were only toxic to cells that expressed leiomodin-1 in the membrane fraction. 

## 2. Results

### 2.1. Leiomodin-1 Is Expressed in Neurons and Astrocytes of the Human CNS

**Figure 1:**
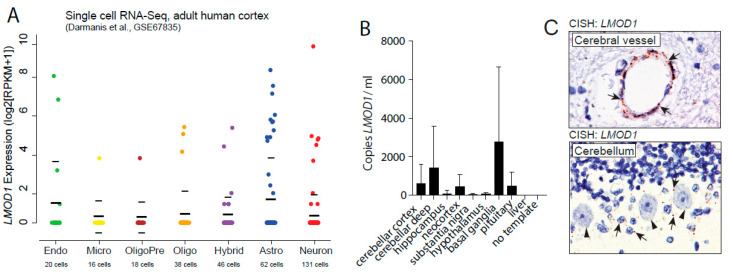
*LMOD1* transcripts are detectable in neurons and glia in the human central nervous system. (**A**) In silico re-analyses of single cell RNA-sequencing data from human cortex show *LMOD1* expression in reads per kilobase of transcript per million (RPKM) (y-axis) in multiple cell types (x-axis) of the central nervous system (CNS) including astrocytes, neurons, and endothelial cells. Thick black bars show mean and light black bars indicate standard deviation. (**B**) Quantitative polymerase chain reaction (qPCR) measuring leiomodin-1 transcripts from post-mortem tissue collected from the cerebellar cortex, cerebellar deep nuclei (cerebellar deep), hippocampus, neocortex, substantia nigra, hypothalamus, basal ganglia, and pituitary. Liver (as low-expressing tissue) and no template control (negative control) were included in the experimental design but not in the analysis. Data are expressed as the mean leiomodin-1 transcript levels (copies per microliter) ± SD from three patients. Data were analyzed by Friedman test, which showed no overall significant difference in leiomodin-1 transcript levels (*p* = 0.30). (**C**) Chromogenic in situ hybridization with *LMOD1* probe set demonstrates the presence of *LMOD1* transcripts in the cerebral vasculature (upper panel) as well as in the Bergmann glia (arrow) of the cerebellum (lower panel). Purkinje cells are indicated with arrowheads.

In silico analyses of single-cell RNA-sequencing data [15] show that leiomodin-1 transcripts are detectable from multiple cell types in the CNS including neurons and astrocytes (Figure 1A). Of the cells analyzed, 22% of astrocytes had detectable *LMOD1* transcripts, whereas a lower proportion of neurons (6.9%) had detectable *LMOD1* transcripts. In addition, in publicly available data from the Allen Brain Atlas, *LMOD1* expression is detectable in human (Figure S1A,B) [16] and murine brain [17] tissue with the highest expression levels in the mesencephalon and epithalamus (https://human.brain-map.org/ and https://mouse.brain-map.org/). A third dataset, from the Genotype-Tissue Expression (GTEx) project, was also examined. Analysis of the GTEx database indicated that *LMOD1* was expressed in multiple regions of the CNS with the highest expression observed in the basal ganglia (Figure S1C). To further assess *LMOD1* expression in the CNS, we performed qPCR amplification of leiomodin-1 transcripts from eight regions (cerebellar cortex, cerebellar deep nuclei, hippocampus, neocortex, substantia nigra, hypothalamus, basal ganglia, and pituitary) of tissue collected during rapid autopsy (Supplementary Table S1). *LMOD1* RNA transcripts were detected from all brain regions (Figure 1B), with the highest levels observed in basal ganglia (mean *LMOD1* copies per µL ± SD: 2818 ± 3845) and cerebellar deep nuclei (mean *LMOD1* copies/µL ± SD: 1478 ± 2098), consistent with the Allen Brain Atlas and GTEx database findings. To localize leiomodin-1 transcripts in tissue sections, we performed RNA in situ hybridization on formalin-fixed paraffin-embedded (FFPE) tissues (Figure 1C). LMOD1 RNA was detectable in both the vasculature as well as in neurons and glia.

**Figure 2:**
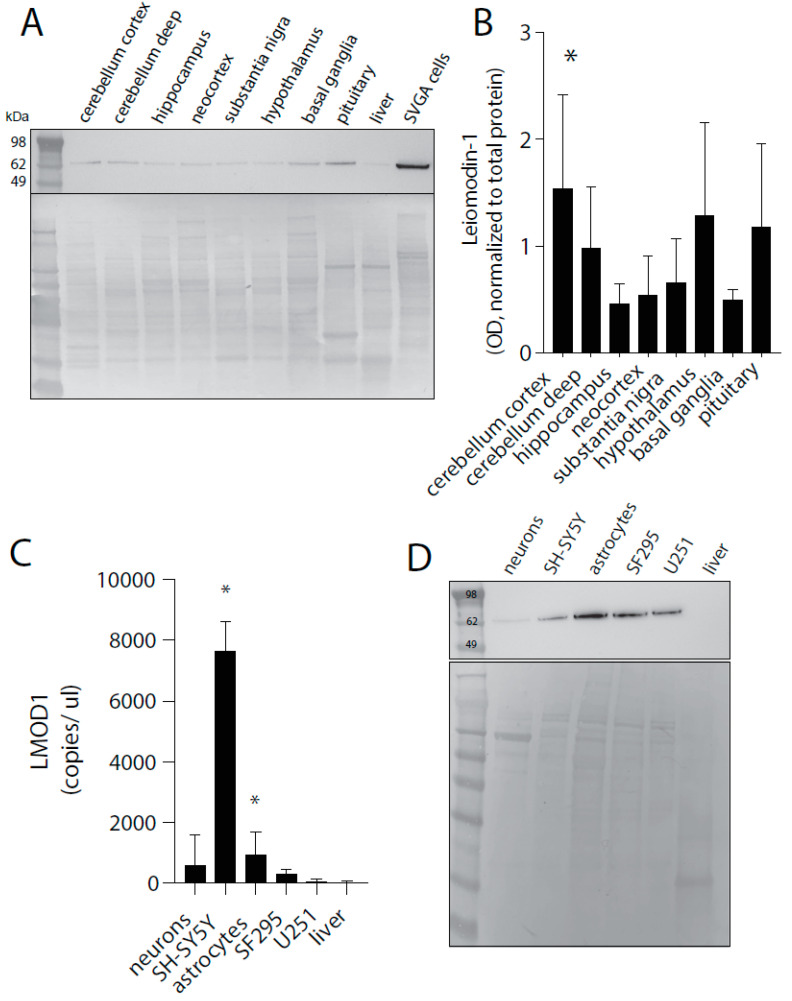
Leiomodin-1 protein is detectable from multiple brain regions and from neurons and glia. (**A**) Representative immunoblot of human brain homogenates with commercial leiomodin-1 antibody (top panel) and total protein (bottom panel) with SVGA cells as a positive technical control. Leiomodin-1 is observed as a single band at approximately 64 kDa. (**B**) Analysis of immunoblotting for leiomodin-1 by optical density (OD). Data are presented as mean OD (normalized to total protein) ± SD from four patients. Data were analyzed by repeated measures- (RM)-ANOVA, which showed an overall significant difference (*p* = 0.0002), and a Dunnett’s correction was applied to correct for multiple comparisons for each region to the neocortex: cerebellar cortex versus neocortex, * = *p* < 0.008. (**C**) Leiomodin-1 transcripts measured by qPCR from neurons, astrocytes, liver, and the cell lines SH-SY5Y, SF295 and U251. Data are expressed as the mean leiomodin-1 transcript levels (copies per microliter) ± SD. Data were analyzed by the Kruskal–Wallis test, which showed an overall significant difference (*p* = 0.01), and a Dunn’s correction for multiple comparisons for cell type as compared to liver. Astrocytes (*p* = 0.01) and SH-SY5Y (0.0007) showed a significant increase in leiomodin-1 transcripts as compared to liver. (**D**) Representative immunoblot for leiomodin-1 from cellular protein extracts (top panel) and total protein (bottom panel).

A recent report [18] has suggested that leiomodin-1 protein is not expressed in the CNS. However, controlled experiments in our laboratory with the same antibody used to make these claims showed a lack of specificity and sensitivity to leiomodin-1 (Figure S2). Although antibody-based detection assays for proteins in human tissue are used widely in research and clinical diagnostics, it is of the utmost importance to validate and optimize antibodies before drawing conclusions based on their immunoreactivity [19,20]. Incomplete validation of biological and chemical reagents is a primary cause of irreproducibility in biomedical investigations [19,21]. Currently, we do not have a validated reagent that shows the high level of specificity and sensitivity our laboratory adheres to for use in FFPE tissues. Therefore, to confirm protein expression, immunoblotting for leiomodin-1 was performed from protein extracts from all CNS regions investigated (Figure 2A). 

Leiomodin-1 protein was detectable in all regions (Figure 2B) with the highest expression in the cerebellar cortex (mean OD + SD, 1.56 + 0.85) as compared to the neocortex (mean OD + SD, 0.56 + 0.35, *p* = 0.008, repeated measures- (RM)-ANOVA with Dunnett’s correction for multiple hypotheses). To further confirm leiomodin-1 expression in neurons and glia, we performed qPCR (Figure 2C) from neurons and astrocytes differentiated in vitro as well as representative cancer cell lines of the CNS including glioblastomas (U251, SF295) and neuroblastoma (SH-SY5Y). Leiomodin-1 was expressed in differentiated neurons and astrocytes but was not detectable in liver—a negative control. *LMOD1* transcripts were significantly enriched in the neuroblastoma cell line SH-SY5Y (mean ± SD = 7701 ± 932.5, *p* = 0.0007, Kruskal–Wallis test) and in astrocytes (mean ± SD = 1003 ± 682.1, *p* = 0.01, Kruskal–Wallis test) as compared to liver (mean ± SD = 30.12 ± 32.45). Protein expression was confirmed using immunoblot analyses (Figure 2D). Together, these findings demonstrate that *LMOD1* transcripts and leiomodin-1 protein are expressed at detectable levels in the human brain and that leiomodin-1 is present in both neurons and glia.

### 2.2. Leiomodin-1 Expression Changes during Development

**Figure 3:**
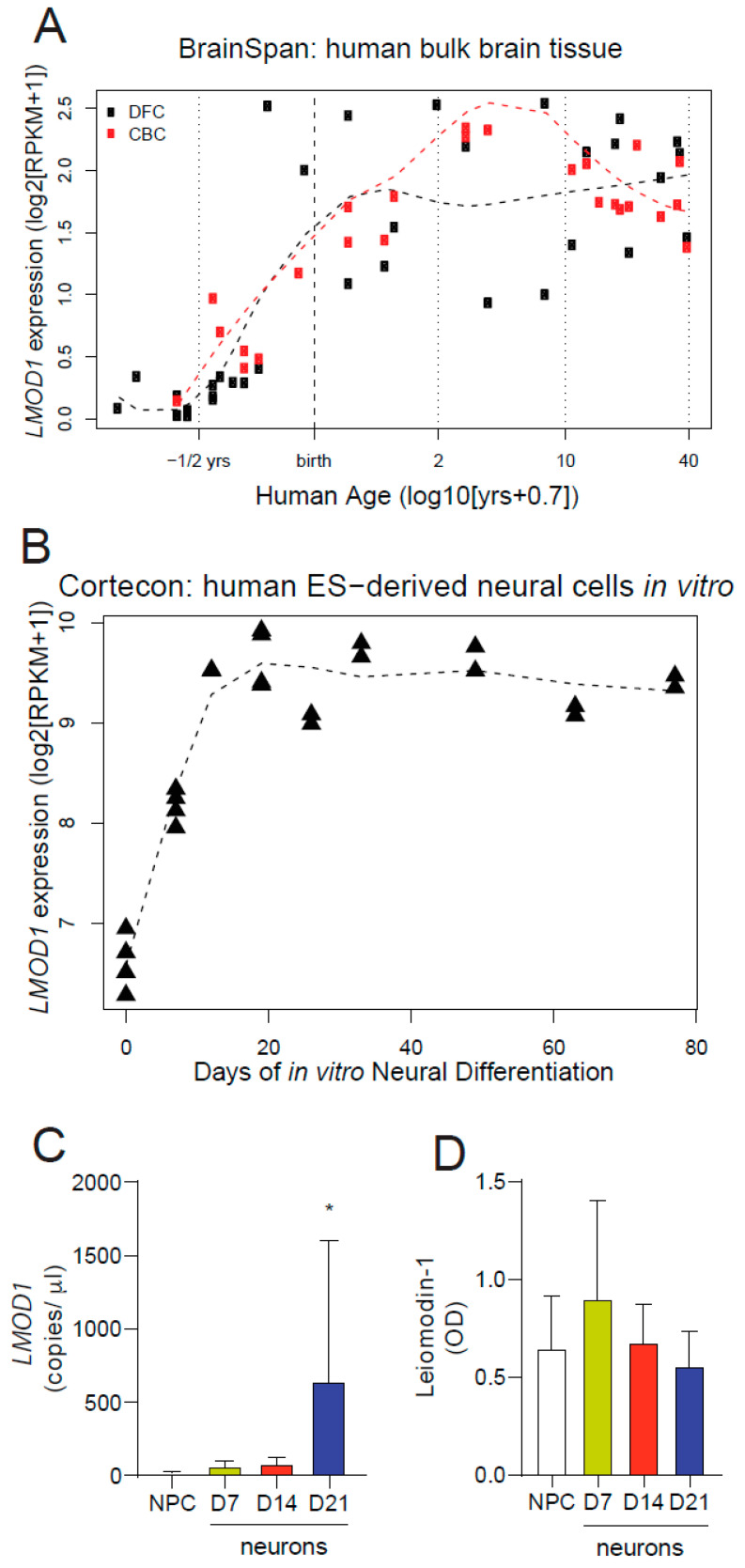
Change in leiomodin-1 expression levels during development. Expression of leiomodin-1 mRNA in (**A**) the BrainSpan database demonstrates that levels increase in the human dorsolateral prefrontal cortex (DFC) and cerebellar cortex (CBC) in utero following peak neurogenesis and plateau near birth; and (**B**) the Cortecon database demonstrates that expression increases as pluripotent cells become dedicated neural progenitors and begin to differentiate towards neurons. (**C**) Quantitative PCR measuring leiomodin-1 transcripts from neural progenitor cells (NPC), newly formed neurons (D7), intermediate neurons (D14) and mature neurons (D21). Data are expressed as the mean leiomodin-1 transcript levels (copies per microliter) ± SD from four independent differentiations. Data were analyzed by the Kruskal–Wallis test, which showed an overall significant difference (*p* = 0.007), and a Dunn’s correction for multiple comparisons for each stage as compared to neural progenitor cells. Only mature neurons showed a significant increase in leiomodin-1 transcripts as compared to neural progenitor cells (* *p* = 0.007). (**D**) Analysis of immunoblotting for leiomodin-1 by optical density (OD). Data are presented as mean OD (normalized to total protein) ± SD from four independent differentiations. Data were analyzed by the Kruskal–Wallis test, which showed no overall significant difference (*p* = 0.71).

To better understand the expression changes of leiomodin-1 during development, we performed in silico analyses of two publicly available datasets. Analysis of the BrainSpan database, a dataset comprised of RNA-Seq data from post-mortem human brain tissue collected at different stages of human brain development [22], showed increased levels of *LMOD1* expression in the prefrontal and cerebellar cortices during the second and third trimester. *LMOD1* expression reached a plateau near birth and remained constant throughout adulthood (Figure 3A). Furthermore, expression analysis of the Cortecon dataset, a database of gene expression in embryonic stem cell-derived developing neural progenitors and neurons [23], demonstrated that *LMOD1* expression increases rapidly as pluripotent cells enter the neural lineage and as neural progenitors differentiate (Figure 3B). To expand on these in silico findings, we examined leiomodin-1 expression in developing neurons in vitro. Neural progenitor cells were differentiated into neurons for either seven days (newly formed neurons, doublecortin (DCX) expressing), 14 days (immature neurons, Tuj1 expressing) or 21 days (mature neurons, microtubule-associated protein 2 (MAP2) expressing) (Figure S3). RNA and protein were collected from neural progenitor cells as well as neurons at each time point. Neural progenitor cells, newly formed neurons, immature neurons, and mature neurons all expressed *LMOD1*, though only mature neurons had higher levels of *LMOD1* transcripts (Figure 3C) as compared to neural progenitor cells (mean *LMOD1* transcripts per µL ± SD for mature neurons, 643.1 ± 957.7 versus neural progenitor cells, 16.93 ± 9.07, *p* = 0.007, Kruskal–Wallis with a Dunn’s correction). No differences in leiomodin-1 protein abundance were detected across the maturational states (Figure 3D). 

### 2.3. Leiomodin-1 Changes Subcellular Localization during Neural Development

**Figure 4:**
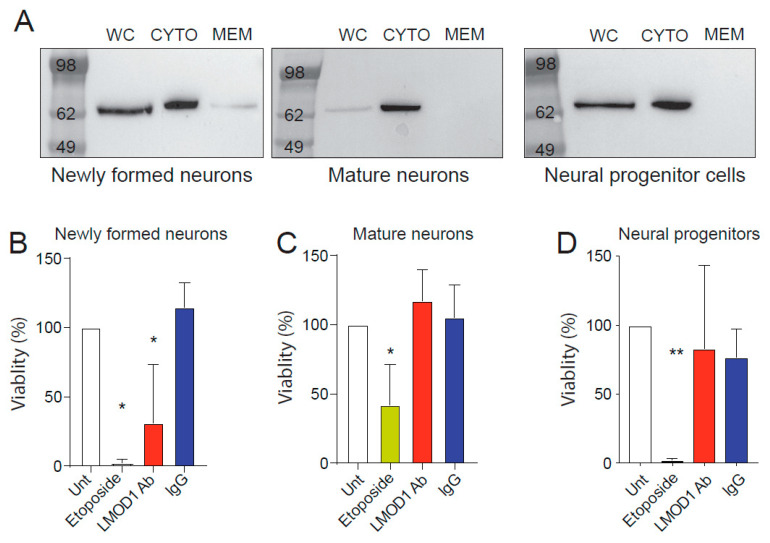
Leiomodin-1 is present in the membrane of newly formed neurons and mediates antibody neurotoxicity. Immunoblots from whole cell lysates (WC), cytoplasmic fractions (CYTO) or membrane fractions (MEM) of (**A**) newly formed neurons, mature neurons and neural progenitor cells demonstrate leiomodin-1 in the membrane fraction of newly formed neurons only. Viability of (**B**) newly formed, seven day-old human neurons, (**C**) mature, 21 day-old neurons and (**D**) neural progenitor cells treated with a commercial leiomodin-1 antibody (LMOD1 Ab) compared to non-specific immunoglobulin G control (IgG) or untreated cells (Unt). Etoposide was used as a positive neurotoxic control. Data shown are percent viability relative to untreated cells. Data were analyzed by one-way ANOVA, which showed an overall significance for each cell type examined (newly formed neurons, *p* = 0.0001, mature neurons, *p* = 0.013, neural progenitor cells, *p* = 0.004). Further comparisons were completed with a Sidak’s correction for multiple hypotheses, comparing etoposide treatment to untreated cells (newly formed neurons, * *p* = 0.0001, mature neurons, * *p* = 0.047, neural progenitor cells, ** *p* = 0.004), leiomodin-1 antibody treatment to untreated cells (newly formed neurons, * *p* = 0.002, mature neurons, *p* = 0.835, neural progenitor cells, *p* = 0.92), non-specific IgG treatment to untreated cells (newly formed neurons, *p* = 0.73, mature neurons, *p* = 0.99, neural progenitor cells, *p* = 0.78), and leiomodin-1 antibody treatment to non-specific IgG treatment (newly formed neurons, *p* = 0.0001, mature neurons, *p* = 0.95, neural progenitor cells, *p* = 0.99).

Leiomodin-1 is an actin nucleating protein [24]. Based on this function, it has been previously suggested that leiomodin-1 is exclusively localized in the cytoplasm of cells. However, prior studies from our group have suggested that antibodies against leiomodin-1 are directly neurotoxic [13]. This led to the hypothesis that leomodin-1 could be present on the membranes of neurons and that this shift in subcellular localization could be linked to neuronal differentiation. To address this question, we examined the subcellular localization of leiomodin-1 at different stages of neuronal differentiation. Membrane and cytosolic fractions from neural progenitor cells, newly formed neurons, and mature neurons were assayed for leiomodin-1 by immunoblotting (Figure 4A and Figure S4A). Fractions from smooth muscle cells, known to express high levels of leiomodin-1, were also examined (Figure S5). Leiomodin-1 was detectable in the cytoplasmic fraction of all cells. Notably, leiomodin-1 was also detectable in the membrane of newly formed neurons whereas neither smooth muscle cells, mature neurons, nor neural progenitor cells showed membranous leiomodin-1 (Figure 4A and Figure S5). Further biochemical analysis of differentiating neurons demonstrates that leiomodin-1 is detectable in the membrane from day six to seven, but that by day eight, leiomodin-1 is no longer present within the membrane fraction (Figure S4B). 

### 2.4. Leiomodin-1 Antibodies Are Toxic to Newly Formed Neurons but Not to Mature Neurons

We had previously shown that leiomodin-1 antibodies from patients with nodding syndrome are neurotoxic [13]. Given the differentiation-dependent localization of leiomodin-1, we tested if leiomodin-1 antibodies would show differential neurotoxicity to neural progenitor cells, newly formed neurons, or mature neurons. Cells were left untreated or treated with either leiomodin-1 antibodies or non-specific immunoglobulin G (IgG) control antibodies for 24 h. Leiomodin-1 antibodies reduced newly formed neurons’ viability (Figure 3B) as compared to cells that received pooled non-specific IgG (mean percent viability ± SD, 31.3% ± 42.2 versus 114.6% ± 17.7, *p* = 0.0001, ANOVA with a Sidak correction for multiple comparisons) and untreated cells (mean percent viability ± SD, 100% ± 0, *p* = 4.20.002). However, leiomodin-1 antibody toxicity was not observed for mature neurons (Figure 3C) (leiomodin-1 antibody viability ± SD = 117.2% ± 22.8, IgG viability = 105.5% ± 23.4, untreated cells viability = 100% ± 0) or for neural progenitor cells (Figure 3D) (leiomodin-1 antibody viability ± SD = 83.3% ± 59.9, IgG viability = 76.9% ± 20.5, untreated cells viability = 100% ± 0). Neural progenitor cells, mature neurons and newly formed neurons were all susceptible to toxicity induced by etoposide. As leiomodin-1 is expressed at high levels in smooth muscle, including in blood vessels, we examined if antibodies to leimodin-1 are toxic to smooth muscle cells (Figure S5). Leiomodin-1 antibodies did not induce toxicity in smooth muscle cells at any time point. Collectively, these findings support the hypothesis that the alterations in the subcellular localization of leiomodin-1 observed during neuronal differentiation are associated with changes in leiomodin-1 antibody-mediated cytotoxicity.

## 3. Discussion

Nodding syndrome is a devastating neurological disease that has occurred in an epidemic fashion in several regions in Africa [3]. In addition to seizures, patients with nodding syndrome also develop cognitive impairments, psychiatric and behavioral disorders, and a lack of maturation and development (reviewed in [25]). Multiple causes for nodding syndrome have been proposed including exposure to munitions or toxins [2], infectious diseases [1,2,8,26], autoimmune responses [13,27], and tauopathy [28]. Despite rigorous investigation, the cause of nodding syndrome remains unclear. While anti-epileptics greatly benefit some patients [29], no cure for this disease is available. An epidemiological association between the parasite *O. volvulus* and nodding syndrome [1,2], as well as other forms of epilepsy [9,30,31,32,33,34,35,36], has been established. However, *O. volvulus* infection is also present in children without nodding syndrome or any form of epilepsy and in areas of the world where nodding syndrome has not been described. However, increased incidence of epilepsy is positively correlated with parasite burden [9], which suggests that this parasite either directly causes neurologic disease or indirectly does so by triggering an immune response. Indeed, we have previously shown that patients with nodding syndrome have antibodies to *O. volvulus* proteins that cross-react with human leiomodin-1 [13], suggesting a potential causative role. However, as leiomodin-1 was previously thought to only be expressed in the cytoplasm, the mechanism by which leiomdodin-1 antibodies caused toxicity was unclear. To further explore the hypothesis that immune responses to leiomodin-1 are a contributing factor to the development of nodding syndrome, we examined the regional expression of leiomodin-1 in the human CNS and the subcellular localization of leiomodin-1 in neural progenitor cells, newly formed neurons, and mature neurons. 

Leiomodin-1 is an actin organizing protein [14,37]. Mutations in *LMOD1* result in megacystis microcolon intestinal hypoperistalsis syndrome, and a complete loss of expression of leiomodin-1 is lethal [38]. Leiomodin-1 was previously described to have expression limited to smooth muscle and the thyroid gland [39,40]. However, here we demonstrate, both at the transcript and protein level, that leiomodin-1 is expressed within the human CNS and that this protein is present in both neurons and glia, albeit at a much lower level than smooth muscle cells. The majority of leiomodin-1 present in the CNS is likely to be from the cerebral vasculature. Patients with nodding syndrome show both cerebral and cerebellar atrophy [4,5] and some patients have stunted growth and delayed sexual maturation, suggesting pituitary-hypothalamic dysfunction [41]. Importantly, the patterns of clinical and pathological changes mirror the expression of leiomodin-1 in our study, with detectable protein expression in the pituitary gland, cerebellum, and hypothalamus. This correlative observation suggests that immune responses to leiomodin-1 could target these regions, resulting in pathology such as cerebellar atrophy as well as abnormal growth and development. 

The hypothesis that leiomodin-1 antibodies could contribute to neurological dysfunction has been controversial as leiomodin-1 was thought not to be expressed in the CNS [42]. Additionally, as leiomodin-1 is expressed in blood vessels, it has been suggested that if antibodies to leiomodin-1 were pathogenic it would result in myopathy or cardiomyopathy [42,43]. This hypothesis assumes that the subcellular localization of leiomodin-1 is uniform in all cell types that express this protein. To explore this hypothesis, we examined the subcellular localization of leiomodin-1 in neurons and in a cell line derived from smooth muscle cells of the cardiovascular system. Upon examination of biochemical fractions from neural progenitor cells and neurons collected during differentiation time courses, we discovered that leiomodin-1 is expressed in the membrane fraction of newly formed neurons. Furthermore, the toxicity mediated by leiomodin-1 antibodies is restricted to the cell populations that contain membranous leiomodin-1. Importantly, leiomodin-1 was not detected in the membrane fraction of smooth muscle cells at any point of differentiation, nor were leiomodin-1 antibodies toxic to these cells. Collectively, these data suggest that leiomodin-1 antibodies are directly interacting with membranous leiomodin-1 in newly formed neurons to initiate a neurotoxic cascade and may thus be contributing to the development of epilepsy. Neurogenesis may occur throughout the lifespan of humans; however, recent data suggest that the vast majority of neurogenesis occurs during infancy and through childhood, with limited detectable new neurons found in adults [44,45]. Disruption of neurogenesis has been directly linked to the development of epilepsy in an animal model [46]. Indeed, it may be that in children with nodding syndrome, the presence of *O. volvulus* antibodies that cross-react with leiomodin-1 disrupts newly formed neurons, leading to the development of epilepsy. This would explain why adults from *O. volvulus* endemic regions may have leiomodin-1 autoantibodies without any neurologic disease. However, children under five also do not typically develop nodding syndrome despite ongoing neurogenesis. This may be explained by the decreased probability of children under five being infected with *O. volvulus*. Studies in Nigeria have demonstrated an increase in the prevalence of *O. volvulus* infection with age [47] and a lack of documented infection in children under one year [48]. Serology studies performed in Uganda have confirmed these findings with children under five years of age demonstrating lower seropositivity as compared to older children [49]. Future longitudinal studies investigating the development of leiomodin-1 antibodies and the development of neurologic diseases including epilepsy could inform this hypothesis. 

This study has several important limitations that future studies may address. Our finding of leiomodin-1 expression in the CNS is based on a limited number of post-mortem samples from our rapid autopsy program. These samples were all from adults. Future studies may examine a greater number of specimens from the pediatric age range. An additional important limitation of this study is that, in our hands, in situ protein detection of leiomodin-1 in human FFPE CNS tissue was not possible. Further development and validation of reagents for use in human FFPE tissues may resolve this issue and future studies could be informative for localization of leiomodin-1 within the human CNS. We also demonstrated that leiomodin-1 is expressed on the membranes of newly formed neurons and that this group of neurons is sensitive to antibody-mediated toxicity. This is similar to other autoimmune epilepsies that have been described in which the immune system targets neuronal surface proteins and disrupts neuronal function [12]. For example, autoantibodies to NMDAR trigger the internalization of this receptor resulting in decreased signal transduction [50] and removal of the autoantibody allows for the recovery of the receptor and abatement of seizure and other clinical manifestations [51,52]. However, the presence of leiomodin-1 antibodies prior to or at disease onset has not been established and it is important not to conflate the findings during symptomatic disease with the conditions present at disease initiation. Indeed, multiple studies have demonstrated that autoantibodies can be detected years before the development of disease symptoms. For example, development of citrullinated protein antibodies often precedes the development of rheumatoid arthritis [53] and islet cell autoantibodies can precedes diabetes [54]. Additionally, the specificity of autoantibodies can change during disease progression. For example, antinuclear and antiphospholipid antibodies were detected in sera collected from individuals from the US military, who subsequently developed systemic lupus erythematosus [55]. However, at disease onset, antibodies against ribonucleoprotein and Smith antigen were present [55]. Therefore, antibodies occurring around the time of onset of symptoms may be markers of disease propagation and not markers of disease initiation (as reviewed in [56]). The studies described in this manuscript do not implicate leiomodin-1 antibodies as an initiating, propagating, or amplifying factor of nodding syndrome. Future prospective studies would be needed to address this hypothesis. Finally, in this study, we did not elucidate the mechanism of leiomodin-1 antibody-mediated toxicity. Additional in vitro investigations to determine this mechanism are warranted as are studies to determine if these antibodies can initiate seizures in microelectrode array models. 

## 4. Materials and Methods 

### 4.1. In Vitro Neuronal and Glial Cell Differentiation

H-9 derived human neural progenitor cells (ENStem-A, EMD Millipore, Burlington, MA, USA) were plated in tissue culture dishes coated with laminin (20 μg/mL) and poly-L-ornithine (10 μg/mL). Neural progenitor cells were maintained in neural progenitor cell complete media (Dulbecco’s modified Eagle medium (DMEM)/F12 knockout supplemented with 2 mM GlutaMax, 2% (*v*/*v*) StemPRO neural supplement, 20 ng/mL epidermal growth factor, 20 ng/mL fibroblast growth factor, and 1% (*v*/*v*) antibiotic/antimycotic solution) at 37 °C with 5% CO_2_ and 90% humidity. Neurons were differentiated with neural differentiation media (Neurobasal DMEM supplemented with 2 mM GlutaMax, 2% (*v*/*v*) B-27 supplement, 1% (*v*/*v*) N2 supplement, 200 nM ascorbic acid, 0.5 mM cyclic adenosine monophosphate, 20 ng/mL brain-derived neurotrophic factor, 20 ng/mL glial cell-derived neurotrophic factor, and 1% (*v*/*v*) antibiotic/antimycotic solution) for up to 21 days at 37 °C with 5% CO2 and 90% humidity. Astrocytes were differentiated in astrocyte media (DMEM with GlutaMax supplemented with 1% (*v*/*v*) N2 supplement, 10% (*v*/*v*) fetal bovine serum (FBS), and 1% (*v*/*v*) antibiotic/antimycotic solution) for 40 days at 37 °C with 5% CO_2_ and 90% humidity.

### 4.2. Cell Lines

SH-SY5Y cells were maintained in Minimum Essential Media (MEM) supplemented with 10% (*v*/*v*) FBS and 1% (*v*/*v*) antibiotic/antimycotic solution at 37 °C with 5% CO_2_ and 90% humidity. SVGA cells, a human glia progenitor cell [57], were maintained in DMEM supplemented with 10% (*v*/*v*) FBS and 1% (*v*/*v*) antibiotic/antimycotic solution at 37 °C with 5% CO_2_ and 90% humidity. SVGA cells were determined previously to express leiomodin-1 and serve as a positive control in these studies. U251 and SF295 cells were maintained in Roswell Park Memorial Institute (RPMI) supplemented with 10% (*v*/*v*) FBS and 1% (*v*/*v*) antibiotic/antimycotic solution at 37 °C with 5% CO_2_ and 90% humidity. HITC6 smooth muscle cells [58] were maintained in M199 media supplemented with 20 mM (4-(2-hydroxyethyl)-1-piperazineethanesulfonic acid) (HEPES), 2 mM L-glutamine, 10% (*v*/*v*) FBS, and 1% (*v*/*v*) antibiotic/antimycotic solution at 37 °C with 5% CO_2_ and 90% humidity. HITC6 cells were differentiated by serum starvation as previously described [58]. SH-SY5Y and SVGA cell lines were provided by Dr. Avindra Nath. The U251 and SF295 cell lines were obtained from the National Cancer Institute, Frederick, MD, USA.

### 4.3. Cell Fractionation

Membrane and cytoplasmic fractions were extracted using the Mem-PER Plus Kit (ThermoScientific, Waltham, MA, USA) per manufacturer’s instructions. All experiments were independently repeated a minimum of three times.

### 4.4. Ethical Approval

Post-mortem tissue samples were collected after approval by the institutional review board at The Johns Hopkins University School of Medicine (CR00032549/IRB00049386).

### 4.5. Immunoblotting

Adult human brain tissues were collected from four neurologically unremarkable patients with cancer diagnoses who elected to undergo rapid autopsy (Supplementary Table S1). Tissues from the pituitary, mammillary body, frontal neocortex, hippocampus, basal ganglia, substantia nigra, cerebellar cortex, and cerebellar deep nuclei were collected. Additionally, liver was collected from two patients. Tissue homogenate extracts were prepared by mechanical homogenization on ice in 1% (v/v) NP-40 lysis buffer and total protein was quantified by bicinchoninic acid assay. Protein extracts were resolved by sodium dodecyl sulfate (SDS)–polyacrylamide gel electrophoresis and transferred to nitrocellulose membranes. Total protein was quantified by Ponceau S staining. Immobilized proteins were analyzed by immunoblotting for leiomodin-1 (1:1000, Abcam #104858, Cambridge, UK). Primary antibody incubations were performed overnight at 4 °C. Horseradish peroxidase (HRP) secondary antibodies (1:5000, Cell Signaling Technologies, Danvers, MA, USA) were incubated at room temperature for one hour. Membranes were developed with enhanced chemiluminescence (Thermo Fisher Scientific). Blots were visualized with Syngene G-Box Chemi HR and images were obtained with GeneSys software (version 1.4.3.0, Genesys, Daly City, CA, USA). Optical density for leiomodin-1 and Ponceau S was determined using ProteinSimple Alpha-View software (version 3.4.0.0, ProteinSimple, San Jose, CA, USA).

Cell lysates and fractions were resolved by SDS–polyacrylamide gel electrophoresis and fractions were examined for markers of cytoplasmic and membrane proteins by immunoblotting. Differentiated cells were analyzed for markers of neuronal differentiation. Primary antibody incubations for glyceraldehyde 3-phosphate dehydrogenase (GAPDH) (cytoplasmic marker, 1:1000, Cell Signaling Technologies, #5174), N-cadherin (membrane marker, 1:1000, Cell Signaling Technologies, #13116), leiomodin-1 (1:1000, Abcam), doublecortin (DCX, 1:1000, Cell Signaling Technologies, #4604), class III beta-tubulin (Tuj1, 1:1000, Cell Signaling Technologies, #5568) and microtubule-associated protein 2 (MAP2, 1:1000, Cell Signaling Technologies, #8707) were performed overnight at 4 °C. HRP-linked secondary antibodies (1:5000, Cell Signaling Technologies) were incubated at room temperature for 1 h. Membranes were developed and imaged as described above.

### 4.6. Immunohistochemistry

Immunohistochemistry was performed for leiomodin-1 on FFPE cell pellets and sections of cerebellum obtained from Johns Hopkins University School of Medicine Department of Pathology tissue repository. Tissue was used from a recent (<one year) post-mortem from a patient with a non-neurologic disease. Prior to immunostaining, sections were deparaffinized in xylene and rehydrated with an ethanol gradient. Heat-induced epitope retrieval was performed by steaming in citrate buffer (pH 6.0) for 20 minutes. Non-specific immunoreactivity was blocked with serum-free protein block (DAKO, Carpinteria, CA, USA) and dual enzyme block (DAKO, Carpinteria, CA, USA). Primary antibodies to leiomodin-1 (2.5 µg/mL for Ab104858 and 5 µg/mL for NBP1-89398) were incubated at room temperature for one hour followed by PowerVision anti-rabbit-HRP (Leica Biosystems, Buffalo Grove, IL, USA) for 30 minutes. Chromogenic development was completed with diaminobenzidine (DAB; Vector Laboratories, Burlingame, CA, USA) and sections were counterstained with hematoxylin (DAKO). Images were captured using a Nikon E400 fluorescence/bright field microscope equipped with a Nikon DXM1200 camera (Nikon Instruments, Melville, NY, USA) and the SPOT Advanced digital imaging software (Diagnostic Instruments, Inc., Sterling Heights, MI, USA). Negative technical controls included sections incubated with no primary antibody (secondary antibody only). 

### 4.7. Immunofluorescence on Smooth Muscle Cells

HITC6 cells grown on glass coverslips were fixed with 4% (*v*/*v*) paraformaldehyde, permeabilized with 0.5% (*v*/*v*) Triton X-100 in phosphate buffered saline (PBS) and blocked with 5% (*v*/*v*) normal goat serum. Coverslips were incubated with leiomodin-1 antibody (Proteintech, #15117-1-AP, rabbit polyclonal, 1:3000) overnight at 4 °C followed by incubation with goat anti-rabbit IgG conjugated with DyLight 549 (Vector Laboratories, 1:400). Actin filaments were stained with Alexa Fluor 488-conjugated phalloidin (Acti-stain 488, Cytoskeleton, #PHDG1, 100 nM in PBS). Cells were mounted with ProLong Glass anti-fade with NucBlue (ThermoFisher Scientific). Fluorescent images were acquired with a Nikon A1R confocal laser scanning system using a 60X oil-immersion objective (numerical aperture 1.4) and 405 nm, 488 nm, and 561 nm lasers, generating up to 9 z-slices with a pixel resolution of 100 nm and z-step size of 250 nm using a Galvano scanner. Maximal intensity projection images were rendered with Nikon NIS-Elements.

### 4.8. RNA In Situ Hybridization

RNA in situ hybridization was performed using the RNAscope 2.5 FFPE Brown Detection Kit (Advanced Cell Diagnostics, Hayward, CA, USA) with probes specific to LMOD1 (NM_012134.2, probe region 892–2078, Advanced Cell Diagnostics, cat no. 444141) as previously described [59]. Slides were imaged using a Nikon E400 fluorescence/bright field microscope equipped with a Nikon DXM1200 camera (Nikon Instruments, Melville, NY, USA) and the SPOT Advanced digital imaging software (Diagnostic Instruments, Inc., Sterling Heights, MI, USA).

### 4.9. Quantitative PCR

Quantitative polymerase chain reaction (qPCR) was performed on total RNA extracted using the RNeasy Plus Mini Kit (Qiagen, Hilden, Germany) from brain regions described above from three individuals, as well as from cultured cell lines, neural progenitor cells, neurons, and astrocytes. RNA quantity and purity were assessed using spectrophotometry and 250 ng from each sample was directly reverse-transcribed into complementary DNA using SuperScript III (Invitrogen, Carlsbad, CA, USA). Transcript levels of leiomodin-1 (*LMOD1*) were amplified from brain regions using primers specific for *LMOD1* (accession number NM_012134, forward sequence: GAAGAACTCCCGTGACCAGCTA, reverse sequence: AGCCTGGTCCTACTGAAGCAGT). Data were acquired on a CFX96 Real-Time PCR Detection System (Bio-Rad, Hercules, CA, USA) with CFX Manager software (V 3.1, Bio-Rad) and compared to a standard curve generated from a quantitative PCR template standard. All primers and standards were obtained from OriGene Technologies (catalog # NM_012134, Rockville, MD, USA). 

### 4.10. Cellular Toxicity

Neural progenitor cells were seeded in black 96-well plates (15,000 cells per well) and maintained for 24 h or differentiated into neurons, as described above, for one, seven or 21 days. HITC6 cells (15,000 cells per well) were maintained for 24 h prior to experimentation. HITC6 cells were differentiated by serum starvation for one, three, or seven days. Cells were left untreated or treated with 1 µg leiomodin-1 immunoglobulin G (IgG), 1 µg rabbit IgG, or 50 µM etoposide in serum free medium (Opti-MEM, Gibco) for 24 h. Assessment of toxicity was performed using a water-soluble cellular reduction sensitive fluorescent dye (Abcam, catalog #112118) for an additional 24 h. Data were obtained on a FLUOStar Omega microplate reader (BMG Labtech, version 1.11, Ortenberg, Germany) with MARS Data Analysis Software (version 1.02, BMG Labtech). All experiments were repeated independently three to five times.

### 4.11. In Silico Analysis

Single cell sequencing data were retrieved from Gene Expression Omnibus (GEO) database, (www.ncbi.nlm.nih.gov/geo; accession no. GSE67835), which contains sequence data from human cortical tissue from eight adults and four embryonic samples ranging from 16 to 18 gestational weeks in age [15]. We retrieved expression data for *LMOD1* from two public data sets: (1) BrainSpan bulk RNAseq data from post-mortem human brain tissue ranging from prenatal to age 40 years (http://www.brainspan.org/) [22] and (2) Cortecon bulk RNAseq data from human embryonic stem cellderived in vitro neural differentiation spanning pluripotency through 77 days of differentiation to post-mitotic neurons (http://cortecon.neuralsci.org/) [23]. All analysis and visualization were carried out in the R statistical language (https://www.r-project.org/). Data were also obtained from the Genotype-Tissue Expression (GTEx) project. The GTEx project was supported by the Common Fund of the Office of the Director of the National Institutes of Health, and by the National Cancer Institute, National Human Genome Research Institute, National Heart, Lung, and Blood Institute, National Institute on Drug Abuse, National Institute of Mental Health, and National Institute of Neurological Disorders and Stroke. The data used for the analyses described in this manuscript were obtained from the GTEx portal (https://gtexportal.org/home/) on 15 October 2020 using Ensembl gene ID ENSG00000163431.12. Data were normalized as previously described (https://gtexportal.org/home/documentationPage#staticTextAnalysisMethods).

### 4.12. Statistical Analysis

Results are presented as mean and standard deviation (SD). For qPCR, results are copies/µL for leiomodin-1 transcripts; for immunoblotting, results are optical density (arbitrary units), and for toxicity results are percent cell viability. Differences in leiomodin-1 transcript expression in brain regions were compared using a Friedman test. Differences in leiomodin-1 protein expression in brain regions were compared using a repeated measures-analysis of variance (RM-ANOVA) with a Dunnett’s correction for multiple comparisons. Differences in toxicity were assessed using an analysis of variance (ANOVA) with a Sidak correction for multiple comparisons. Differences in leiomodin-1 transcript expression in neural progenitor cells, differentiating neurons, astrocytes, and cell lines were assessed using a Kruskal–Wallis test with a Dunn’s correction for multiple comparisons. Differences in leiomodin-1 protein expression were compared using a RM-ANOVA with a Dunnett’s correction for multiple comparisons. For all statistical tests, a *p* < 0.05 was considered statistically significant. All statistical analyses were performed with GraphPad Software 6.01 (GraphPad Software, San Diego, CA, USA) or SAS version 9.3 (SAS Institute, Cary, NC, USA).

## 5. Conclusions

In summary, we demonstrate expression of leiomodin-1 transcripts and proteins in the CNS, show that leiomodin-1 is present in the membrane fraction of newly formed neurons, and provide the first evidence of selective immune targeting of newly formed neurons by leiomodin-1 antibodies. Our data may provide an explanation for the age restriction of nodding syndrome to the pediatric population and suggest further areas of research that are critical for understanding the pathobiology of autoimmune epilepsies. 

## Supplementary Materials

The following are available online at https://www.mdpi.com/2076-0817/9/12/1036/s1, Figure S1: LMOD1 transcripts are detectable in human brain, Figure S2: Evaluation of leiomodin-1 antibodies for use in immunoblotting and IHC in FFPE tissues, Figure S3: Quantitative analysis of markers of neuronal cell differentiation expressed by in vitro differentiated neurons, Figure S4: Immunoblots from cell fractions from differentiating neurons, Figure S5: Leiomodin-1 is not present in the membrane of arterial smooth muscle cells, Table S1: Demographics of patient samples used in post-mortem analyses.
