# Supplementary material for: Putative Autoantigen Leiomodin-1 Is Expressed in the Human Brain and in the Membrane Fraction of Newly Formed Neurons
Source: Pathogens. 2020 Dec 10;9(12):1036. doi: 10.3390/pathogens9121036 (PMC7763904; doi:10.3390/pathogens9121036)
Supplement: Supplementary file 1 [file pathogens-09-01036-s001.pdf]

Supplemental Appendix

Supplemental Table 1. Demographics of patient samples used in post-mortem analyses.

Supplemental Figure 1. *LMOD1* transcripts are detectable in human brain.

Supplemental Figure 2. Evaluation of leiomodin-1 antibodies for use in immunoblotting and IHC in FFPE tissues.

Supplemental Figure 3. Quantitative analysis of markers of neuronal cell differentiation expressed by *in vitro* differentiated neurons.

Supplemental Figure 4. Immunoblots from cell fractions from differentiating neurons.

Supplemental Figure 5. Leiomodin-1 is not present in the membrane of arterial smooth muscle cells.

Supplemental Table 1. Demographics of patient samples used in post-mortem analyses.

Sample Number	Age (years)	Sex	Cause of death	Post mortem interval (hours)
1	75	M	prostate carcinoma	4
2	65	M	neuroendocrine carcinoma	5
3	30	M	idiopathic cardiomyopathy	11
4	60	F	melanoma	4

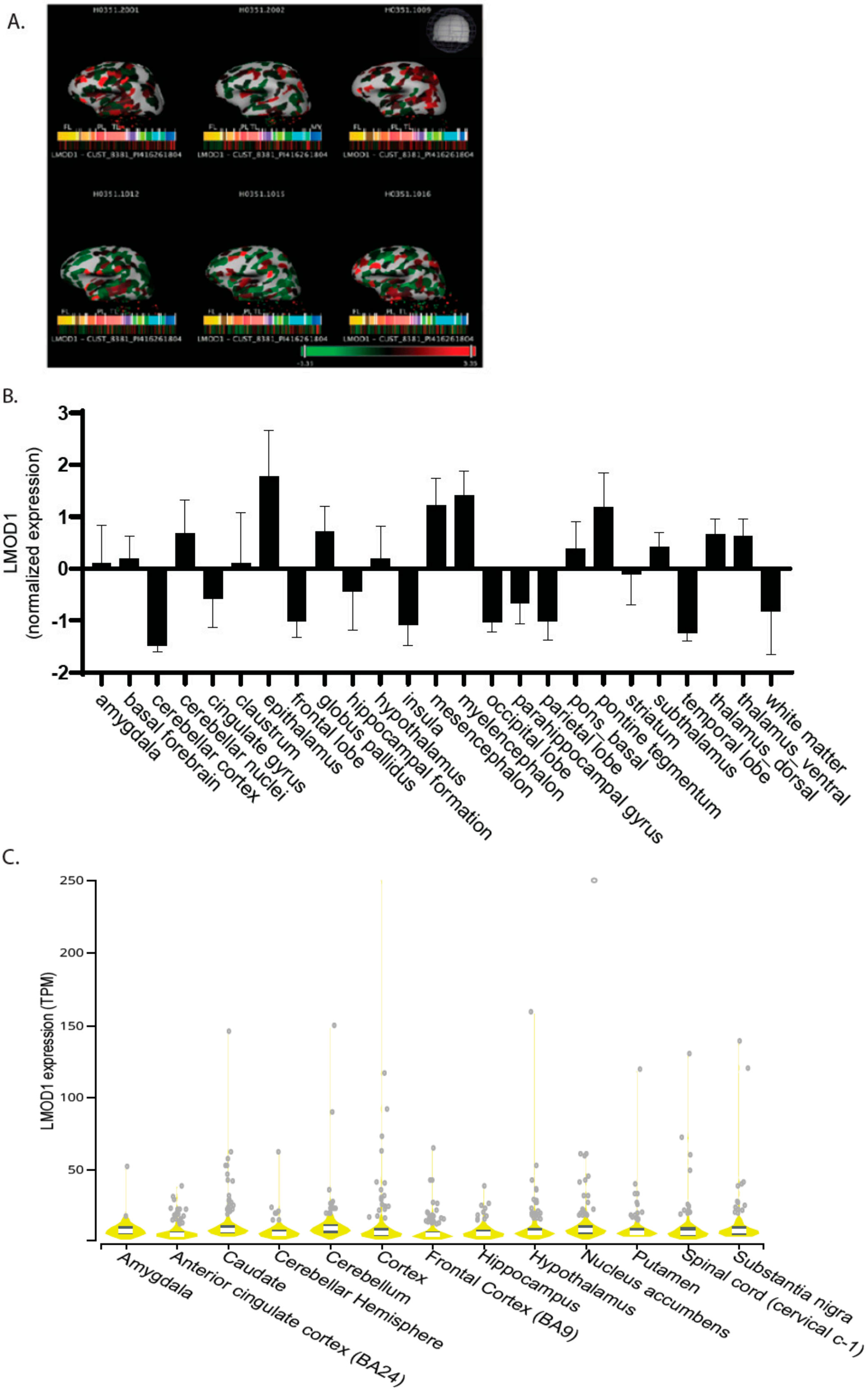
A.
B.
LMOD1
(normalized expression)
3
2
1
0
-1
-2
amygdala
basal forebrain
cerebellar cortex
cerebellar nuclei
cingulate gyrus
claustrum
epithalamus
frontal lobe
globus pallidus
hippocampal formation
hypothalamus
insula
mesencephalon
myelencephalon
occipital lobe
parahippocampal gyrus
parietal lobe
pons basal
pontine tegmentum
striatum
subthalamus
temporal lobe
thalamus_dorsal
thalamus_ventral
white matter
C.
LMOD1 expression (TPM)
250
200
150
100
50
Amygdala
Anterior cingulate cortex (BA24)
Caudate
Cerebellar Hemisphere
Cerebellum
Cortex
Frontal Cortex (BA9)
Hippocampus
Hypothalamus
Nucleus accumbens
Putamen
Spinal cord (cervical c-1)
Substantia nigra

Figure S1. *LMOD1* transcripts are detectable in human brain. (A) Screenshots of topographical heatmaps of *LMOD1* expression in human (18) brain from the Allen Institute demonstrate localized expression of *LMOD1* in the human neocortex. (B) Transcript expression of *LMOD1* in different regions of the brain measured by microarray was downloaded from The Allen Institute. Normalized *LMOD1* gene expression (y-axis) is shown for multiple brain regions (x-axis). (C) *LMOD1* expression from bulk RNA sequencing of different CNS regions was downloaded from the GTEx database. *LMOD1* expression is shown in transcript per million (TPM, y-axis) plotted for different brain regions and the spinal cord (x-axis).

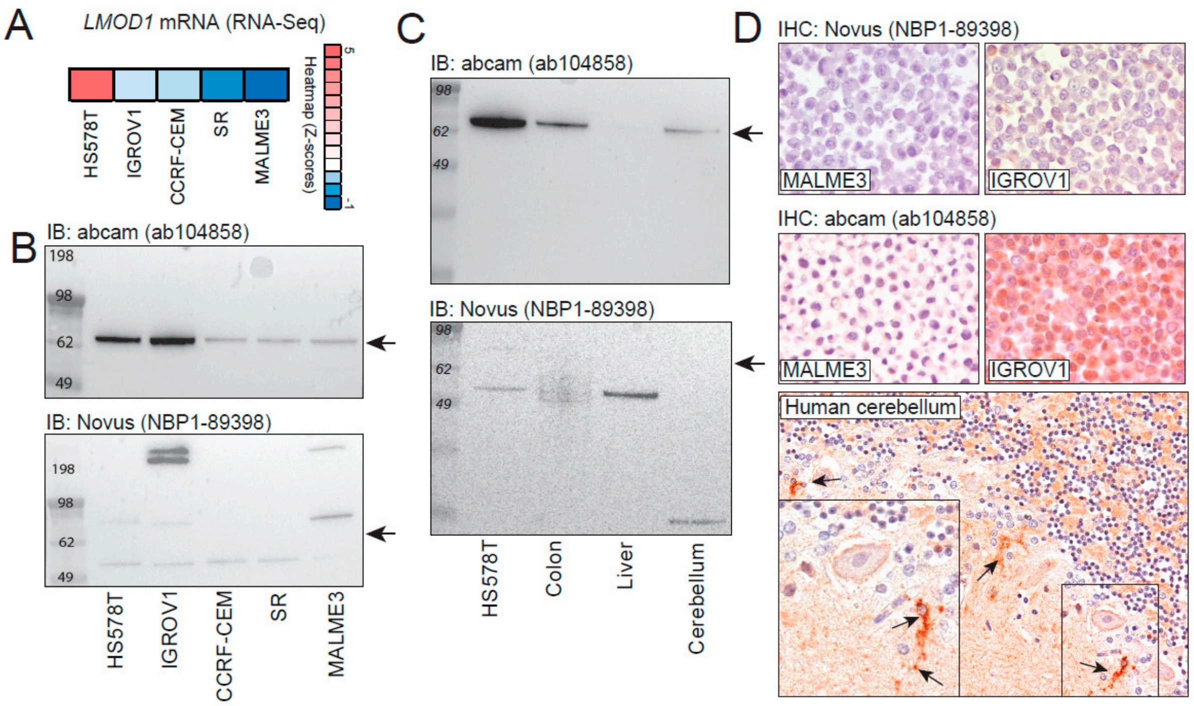

Figure S2. Evaluation of leiomodin-1 antibodies for use in immunoblotting and IHC in FFPE tissues. (A) Heat map of LMOD1 mRNA expression in human cancer cell lines. (B) Immunoblots of HS578T, IGROV1, CCRF-CEM, SR, and MALME3 cell line lysates probed with Ab104858 (upper panel) and NBP1-89398 (lower panel) leiomodin-1 antibodies. Note that Ab104858 demonstrates a single band at approximately 64 kDa, the molecular weight of leiomodin-1 (arrowhead), whereas the NBP1-89398 antibody shows multiple bands at various sizes, but no bands at 64 kDa. (C) Immunoblot of HS578T, human colon tissue (high *LMOD1* mRNA), liver (low/no *LMOD1* mRNA), and cerebellum probed with Ab104858 (upper panel) shows a single band at 64kDa in colon and cerebellum, but not in liver tissue. Note that under the same conditions, NBP1-89398 (lower panel) demonstrates multiple non-specific bands and no reactivity in colonic tissue lysates. (D) Immunohistochemistry on FFPE cell pellets of IGROV1 (*LMOD1* high) and MALME3 (*LMOD1* low) cells shows a lack of immunoreactivity of NBP1-89398 (upper panel) and a strong correlation of immunoreactivity and mRNA expression for the Ab104858 antibody (middle panel).

Immunohistochemistry on sections of cerebellum using the Ab104858 antibody (lower panel) demonstrates immunoreactivity in the gray matter including Bergmann glia (indicated by arrows), however, notable and widespread background staining is apparent in the granule cell neuropil and the neuropil of the molecular layer. Original magnification 40x.

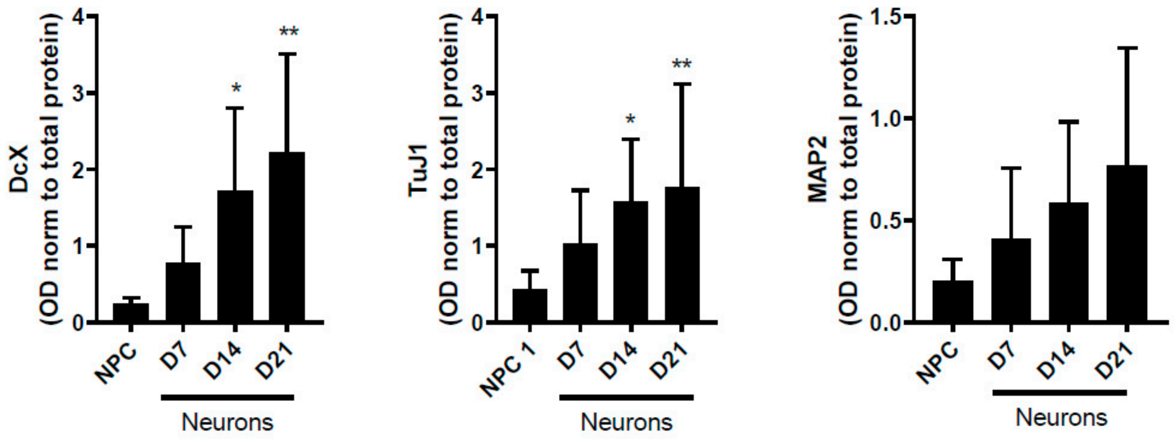

<u>Figure S3. Quantitative analysis of markers of neuronal cell differentiation expressed by *in vitro* differentiated neurons.</u> Human derived neural progenitor cells (NPC) were differentiated for seven, 14, and 21 days in neuronal media. Protein was harvested, resolved by SDS-PAGE, and transferred to nitrocellulose. Membranes were immunoblotted for Dcx (newly formed neurons), TuJ1 (intermediate neurons), and Map2 (mature neurons). Protein levels were quantified by optical density (OD) and normalized to total protein. Data presented are mean OD (arbitrary units) ± SD for four independent experiments. Data were analyzed using a RM-ANOVA with a Dunnett's correction for multiple hypotheses, comparing each differentiated cell type to NPC, * indicates P<0.05, ** indicated P<0.01. Analysis demonstrates correct characterization for each cell type differentiated.

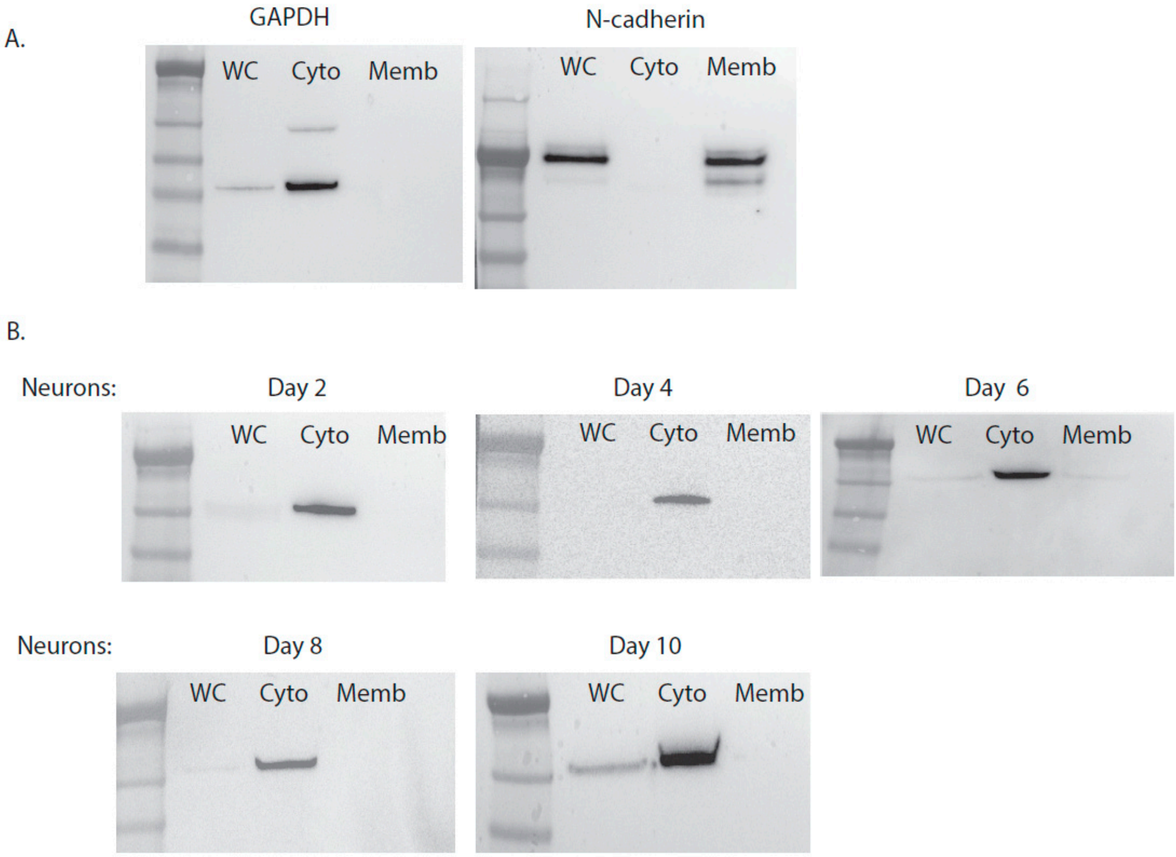

<u>Figure S4. Immunoblots from cell fractions from differentiating neurons.</u> (A) Representative immunoblots showing clear separation of membrane and cytosolic fractions. Whole cell (WC), cytoplasmic (Cyto) and membrane (Memb) lysates were probed with GAPDH as a marker of the cytoplasmic fraction and N-cadherin as a marker of the membrane fraction. No cytoplasmic marker is observed in the membrane fractions and every fractionation was quality controlled in this manner. (B) Immunoblot for leiomodin-1 in the membrane and cytoplasmic fraction for cells differentiated in neuronal media for two, four, six, eight and 10 days. At day six cells express leiomodin-1 in the membrane that is absent at all other time points.

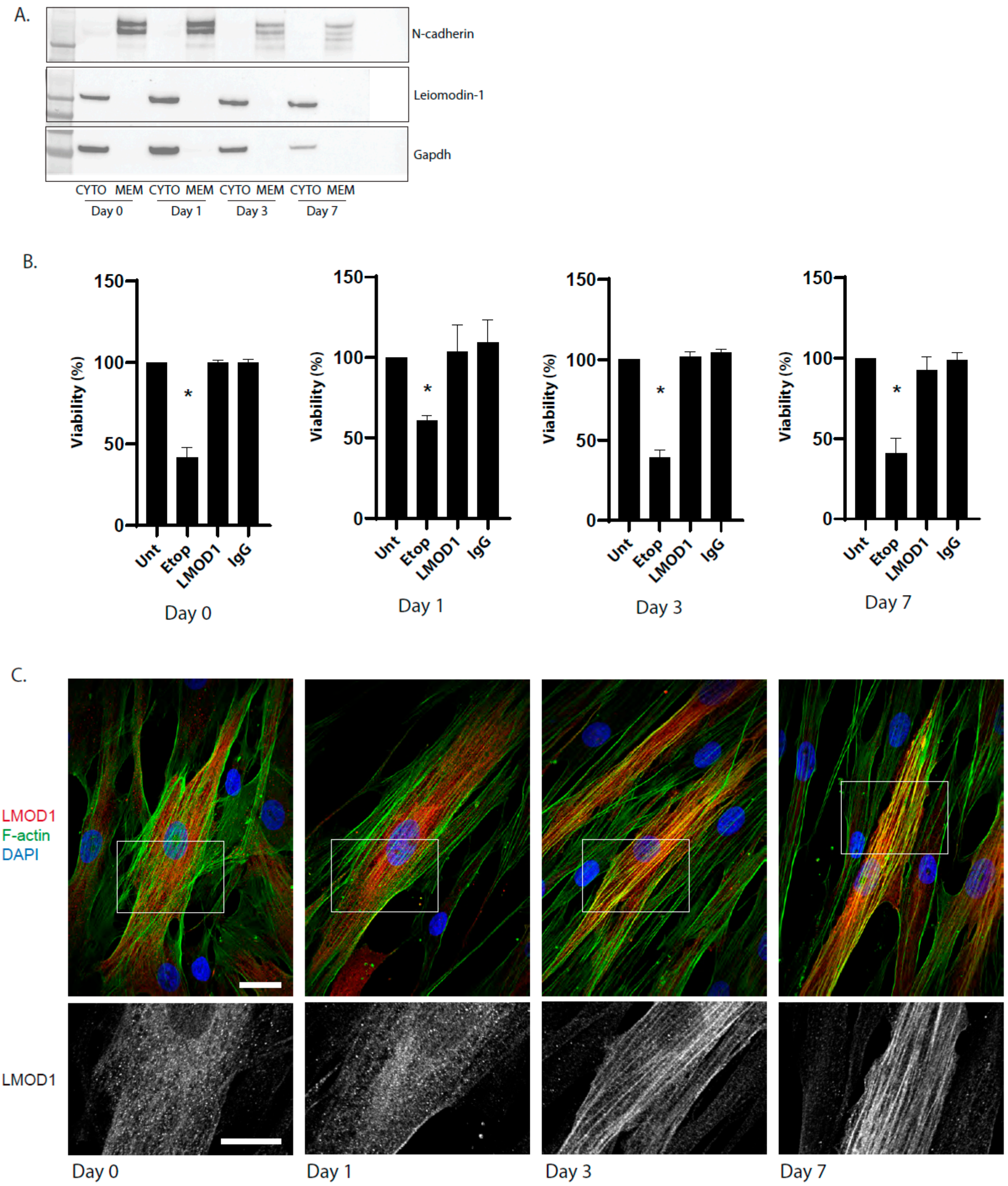

Figure S5. Leiomodin-1 is not present in the membrane of arterial smooth muscle cells. (A) Immunoblots from cytoplasmic (CYTO) or membrane fractions (MEM) of HITC6 cells that were undifferentiated (Day 0) or differentiated for one (Day 1), three (Day 3), or seven (Day 7) days demonstrate that leiomodin-1 is not in the membrane fraction. GAPDH is only present in the cytoplasmic fraction and N-cadherin is present in the membrane fraction confirming the validity of the biochemical fractionation. (B) Viability

of undifferentiated (Day 0), Day 1, Day 3, and Day 7 treated with a commercial leiomodin-1 antibody (LMOD1) compared to non-specific IgG control (IgG) or untreated cells (Unt). Etoposide was used as a positive toxic control. Data shown are percent viability relative to untreated cells. Analyses (ANOVA with Sidak's correction for multiple hypotheses) revealed that only etoposide treated cells showed a reduction in viability (mean ± SD, Day 0: 41.6 ± 6.3, $P = 0.0001$; Day 1: 60.9 ± 2.8, $P = 0.003$; Day 3: 39.3 ± 4.5, $P= 0.0001$; Day 7: 40.8 ± 9.2, $P = 0.0001$). Neither leiomodin-1 (mean ± SD, Day 0: 99.8 ± 1.6, $P = 0.99$; Day 1: 103.5 ± 16.9, $P = 0.99$; Day 3: 102.1 ± 2.8, $P= 0.81$; Day 7: 92.4 ± 8.6, $P = 0.50$) or IgG (mean ± SD, Day 0: 99.9 ± 2.4, $P = 0.99$; Day 1: 109.5 ± 13.7, $P = 0.71$; Day 3: 104.4 ± 2.0, $P= 0.21$; Day 7: 98.5 ± 5.2, $P = 0.99$) treated cells showed decreases in cell viability. (C) Immunofluorescence for leiomodin-1 (red) and F-actin (green) show punctate staining of leiomodin-1 on Day 0 and Day 1. On Day 3 and Day 7, as SMCs shift to a more contractile phenotype, LMOD1 appears to co-localize with the F-actin microfilament bundles. The nuclei is shown with DAPI. Lower panels correspond to the framed regions of interest in upper panels. Scale bar, 20 µm.
